# Synthesis and characterization of core-shell mussel inspired magnetic molecularly imprinted polymer nanoparticles for the solid phase extraction of levofloxacin in human plasma

**DOI:** 10.1186/s13065-025-01641-9

**Published:** 2025-10-22

**Authors:** Aya A. Mouhamed, Kareem Orensa, Maria Osama Mekhail, Noha I. Abdelaziz, Amr M. Mahmoud, Dina A. El Mously

**Affiliations:** 1 Pharmaceutical Analytical Chemistry Department, Faculty of Pharmacy, Cairo University, El-Kasr-El Aini Street, Cairo, 11562 Egypt; 2 School of Pharmacy, Newgiza University, Km. 22 Cairo-Alex Road, P.O. Box 12577, Giza, Egypt; 3 Faculty of Pharmacy, Cairo University, El-Kasr-El Aini Street, Cairo, 11562 Egypt

**Keywords:** Magnetic solid phase extraction, Therapeutic drug monitoring, Mussel-inspired polymers, Levofloxacin, Auto-polymerization

## Abstract

Determination of pharmaceuticals in biological matrices is crucial for pharmaceutical development, toxicological studies, and therapeutic drug monitoring. Accurate quantification of drugs in plasma requires reliable analytical techniques; however, the complexity of biological fluids hinders direct analysis without an effective sample preparation step. In this study, Fe₃O₄ was synthesized using mussel-inspired magnetic molecularly imprinted polymer nanoparticles (MIP NPs) through a single-step auto-polymerization process with levofloxacin as the template and methyldopa as the monomer. The integration of Fe₃O₄ core synthesis, self-polymerization, and molecular imprinting in a single step provides a unique and streamlined methodology, reducing fabrication complexity and time. The prepared Fe₃O₄@MIP NPs were successfully applied to extract levofloxacin from spiked human plasma. UV–spectroscopy studies confirmed selective recognition, binding efficiency, and optimization of recovery conditions, achieving approximately 93.5% recovery. The Fe₃O₄@MIP NPs demonstrated significant imprinting capability and high adsorption capacity. This approach offers a novel and cost-effective platform for rapid, selective drug extraction from complex biological matrices, with direct clinical relevance for therapeutic drug monitoring of levofloxacin in multidrug-resistant tuberculosis, particularly suited for resource-limited settings.

**Supplementary Information:**

The online version contains supplementary material available at 10.1186/s13065-025-01641-9.

## Introduction

Sample preparation is one of the most crucial steps when working with complex biological samples, as it removes the multitude of components that may hamper the accurate and precise analysis of the analyte(s) of interest [1].

The diverse nature of biological fluids like serum, urine, and saliva that are constituted of lipids, proteins, impurities, and degradation products requires equally complicated and time-consuming sample clean-up processes [2]. Therefore, reducing the preparation time and simplifying these processes have been important to address the major drawbacks that make these processes consume around two-thirds of the total analysis time and make them a major source of error in the overall analytical process [3–5]. Several methods have been explored in the past to limit this effect [6–8]. The fundamental idea behind all sample preparation techniques is to create a sample from a real matrix that is appropriate for analysis using a separation or other analytical methods [9]. Commonly used extraction techniques are protein precipitation (PPT), liquid/liquid extraction (LLE), and solid-phase extraction (SPE) [10].

As an innovative type of solid-phase extraction (SPE), magnetic solid-phase extraction (MSPE) has gained widespread popularity for its superiority in terms of overcoming the disadvantages of traditional SPE [11]. In MSPE, the use of magnetic adsorbents directly in sample solutions enhances the contact area between adsorbents and analytes, increasing their extraction efficiency. Furthermore, it overcomes the limitations related to adsorbent packing, such as high pressure and packed bed clogging [10]. MSPE is a notably simple, cost-effective, and environmentally friendly technique, as the retrieval of the magnetic adsorbents is primarily simplified by the use of an external magnet rather than the requirement for filtration and centrifugation and is also possible to be recycled and reused [12, 13] .

Magnetic adsorbents, which usually consist of magnetic carriers, are vital to MSPE because of their direct influence on extraction efficiency and simultaneous evaluation of the sensitivity and selectivity of the method [14]. Thus, the use of magnetic nanoparticles (MNPs), such as Fe, Co, and Ni, especially the Fe_3_O_4_ and Fe_2_O_3_ NPs is employed in MSPE and contributes to the superior features of this technique [15]. The application of the MNPs requires both chemical and physical stability, which are directly affected by the choice of synthesis method. Among the popular techniques are co-precipitation, sol-gel, hydrothermal, microwave and sonochemical synthesis [12, 16]. Additionally, the synthesized MNPs exhibit no magnetization once the magnetic field is removed because of their superparamagnetic properties, making them appropriate for in vivo applications [17]. However, the need for pre-concentration of analytes, as well as their selective and sensitive separation and molecular identification, remains a considerable challenge [18–20] .

From this perspective, the development of molecularly imprinted polymers (MIPs), which are synthetic polymers that reshape their surface according to the molecular structure of the target molecule, became a promising approach to overcome the challenges facing MNPs [21]. These tailor-made NPs enable the selective and effective targeting of certain molecules for separation, purification, and isolation in complicated mixtures through the paired application of MIPs and magnetic core-shell imprinting (MMIP NPs) [22]. In addition, the functionalization of MNPs using the surface imprinting technique is expected to enhance the heterogeneous distribution of binding sites, low binding capacity, and slow binding kinetics that are the main disadvantages of MIPs while maintaining their effective and appealing role as sorbents in SPE [3, 23].

Recent advances have further strengthened the relevance of MSPE and MIP-based strategies in biological analysis [24]. The preconcentration of fluoroquinolones (FQs) including levofloxacin (LFX) has been successfully demonstrated using magnetic nanomaterials, emphasizing their utility in therapeutic drug monitoring. Recent work on magnetic molecularly imprinted polymers (MMIPs) highlights optimization of core–shell synthesis, imprinting efficiency, and adsorption performance, leading to improved selectivity in complex biological fluids [25]. In parallel, mussel-inspired polymers such as poly(methyldopa) have been increasingly applied as functional monomers for imprinting, yielding highly selective electrochemical sensors and biosorbents for drugs and biomolecules in plasma samples. These studies not only validate the feasibility of MMIPs for antibiotic monitoring but also underline the potential of PMD coatings to address issues of binding heterogeneity, film stability, and non-specific adsorption.

To this end, the novel use of self-polymerizing adhesive proteins that mimic the ability of mussel adhesive proteins (MAPs), which are excreted by sea mussels, to create powerful adhesive bonds to a variety of inorganic and organic objects in moist settings has gained much interest [26, 27]. In alkaline medium, dopamine readily undergoes oxidative self-polymerization, initiating with its conversion to dopaminequinone, followed by intramolecular cyclization and subsequent oxidation/polymerization steps. Throughout this process, both covalent and non-covalent interactions contribute to the progressive formation of the polydopamine (PDA) matrix. The use of PDA films provides ease of use and due to their bioinspired nature, a biocompatible molecularly imprinted polymer coating on nanoparticles that can be used for a multitude of applications in analysis [28, 29]. This work demonstrates the innovative use of poly-(methyldopa) (PMD), a mussel-inspired coating material which is used in molecular imprinting applications. Compared with conventional dopamine or levodopamine coatings, PMD offers enhanced film stability, improved functional group availability, and greater biocompatibility, which collectively improve the robustness and selectivity of the imprinted layer. This innovation allows for more efficient molecular recognition, reduced nonspecific adsorption, and superior extraction performance under complex biological conditions. Thus, the designed Fe₃O₄@MIP NPs provide both material-level advantages (ease of synthesis, eco-friendliness, reusability) and application-level novelty (highly selective enrichment of LFX from plasma), positioning this work as a distinct advancement over existing MSPE-MIP systems.

Monitoring the level of antibiotics in bodily fluids is crucial to improve therapeutic outcomes and avoid the development of bacterial resistance [30]. LFX belongs to the FQs, the cornerstone of treating multi-drug-resistant tuberculosis (MDR-TB) [31]. They exhibit concentration-dependent activity against both Gram-positive and Gram-negative bacteria [32]. LFX is the drug of choice, specifically in low-middle-income countries like Egypt, for treating MDR-TB due to its affordability and availability [33, 34]. The sub-therapeutic systemic concentrations believed to be the root cause of LFX- developed resistance make selective and rapid therapeutic drug monitoring (TDM) of plasma levels a necessity for optimum FQ treatment [35]. Numerous chromatographic techniques, such as HPLC with fluorescence and ultra-violet determination have been employed [32, 36, 37]. Even though the spectroscopic techniques are sensitive and selective [38], they still require rigorous sample pretreatment for TDM, necessitating the need for a cost-effective, rapid, and selective pretreatment method.

In this study, we report a unique, simple technique for creating a molecularly imprinted polymer layer employing mussel-inspired poly-(methyldopa) (PMD) LDP on magnetic Fe_3_O_4_ NPs to remove and enrich LFX from human plasma. The morphology and composition of Fe_3_O_4_@MIPs and Fe_3_O_4_@NIPs were characterized using Zeta-sizer, XPS, SEM, EDX, XRD and FTIR. Through binding studies, the adsorption capacity and magnetic separation capability were evaluated using LFX in the presence of ciprofloxacin and vancomycin, which allowed for the examination of the selectivity and molecular recognition capabilities of Fe_3_O_4_@MIPs for LFX. Additionally, the selective sorbents Fe_3_O_4_@MIPs were effectively used to extract and isolate LFX from human plasma samples that had been spiked. A quick and accurate colorimetric method was then used to determine the level of LFX in the plasma.

## Experimental

### Materials

Levofloxacin, ciprofloxacin, dopamine, and methyl-dopa were all supplied by Amoun Pharmaceuticals, Egypt (LFX was labelled as having a purity greater than 99%). Vancomycin was supplied by Global Napi Pharmaceuticals, Egypt. Human plasma was obtained from VACSERA, Egypt. Glacial acetic acid, sodium chloride, sodium hydroxide and aminomethane (C_4_H_11_NO_3_, Tris) were acquired from Piochem (Cairo, Egypt). Acetonitrile was acquired from Sigma Aldrich, Germany. Double distilled-deionized water originated from the Milli-Q instrument (Millipore Corporation, France) was used in all the experiments. All other chemicals and reagents utilized were of analytical grade.

### Instrumentation

The size and charge of the synthesized nanoparticles were defined utilizing a zetasizer 6.12 (Nano-ZS, Malvern Instruments Ldt., Malvern, UK). X-ray photoelectron spectroscopy (XPS) measurements were conducted using a K-ALPHA system (Thermo Fisher Scientific, USA) equipped with a monochromatic Al Kα radiation source (energy range: 10–1350 eV). The measurements were performed with a spot size of 400 μm under an ultrahigh vacuum pressure of approximately 10⁻⁹ mbar. A pass energy of 200 eV was used for the full spectrum, while 50 eV was used for narrow scans. Scanning electron microscopy (SEM), (The Quanta FEG-250) was utilized to measure and characterize the various morphologies of the synthesized nanoparticles. Fourier-transform infrared (FTIR) spectroscopy was carried out using an infrared spectrophotometer (Shimadzu 435, Kyoto, Japan). Powder X-ray diffraction (PXRD) patterns were measured on PANanalytical X-Ray Diffraction equipment model X׳Pert PRO with Secondary Monochromator, Cu-radiation (λ=1.542Å) at 45 K.V., 35 M.A. and scanning speed 0.04o/sec. were used. The diffraction peaks between 2θ = 2o and 60o, corresponding spacing (d, Å) and relative intensities (I/Io) were obtained. A double-beam UV-Vis spectrophotometer model UV-1900i from Shimadzu, Japan, was used to measure the levels of LFX in the solutions. For the drug assays under examination, the variable wavelength UV-Vis detector was operated at maximum.

### Preparation of magnetic molecularly imprinted polymer nanoparticles (MMIP NPs) for levofloxacin extraction

#### Fabrication of core-shell Fe_3_O_4_ NPs

The magnetic nanoparticles (Fe_3_O_4_) MNPs were prepared by the co-precipitation technique as reported previously [3]. Briefly, 100 mL of FeCl_3_ (40 mmole) solution was mixed with 100 mL of FeSO_4_ (mmole) solution and purged with N_2_ for 15 min. Then, 100 mL of NaOH (300 mmole) solution was added drop by drop under vigorous stirring. Furthermore, oleic acid (2.0 mL) was added, and the mixture was heated to 80 °C for 1 h. Finally, the magnetic nanoparticles’ black precipitate was collected using an external magnet, washed three times with water and methanol to eliminate any unreacted substrates, and then dried at 70 °C.

#### Fabrication of self-polymerizing Fe_3_O_4_@MDA@MIP and Fe_3_O_4_@MDA@NIPs

50 mg of the prepared magnetic Fe_3_O_4_ NPs were dispersed in 20 mL of 0.1 M tris buffer (pH 8.5) by sonication for 10 min until a homogenous suspension is obtained. At the weakly alkaline pH, 100 mg of the self-polymerizing methyldopa, and 83.3 mg of the template molecule LFX were added followed by agitation on a vortex mixer, and the mixture was left under stirring for 24 h at room temperature to convert the template-monomer complex to MIP. The resultant Fe_3_O_4_@MIP NPs were collected by an external neodymium magnet. The template molecule LFX was extracted from the polymer with 10% acetic acid, this was repeated four times to ensure complete elution. Finally, the NPs were rinsed with distilled water. The same process was repeated to manufacture the non-imprinted Fe_3_O_4_@NIP NPs in the absence of the template LFX.

### Analysis techniques

#### Spectrophotometric method for binding tests

A standard LFX solution (0.1 mg/mL) was prepared and scanned between 200 and 400 nm to determine the wavelength of maximal absorption. A calibration curve was established utilizing five different concentrations of standard LFX solution at the maximum wavelength of 298.0 nm [39].

### Binding experiments for the optimization of molecularly imprinted solid phase extraction procedure

Different conditions were initially examined for optimization of the extraction of LFX. The effect of pH on the adsorption capacity of Fe_3_O_4_ MIP NPs for LFX was investigated over a pH range of 5.0 to 8.0. To ensure the Fe_3_O_4_ @MIP NPs were free from any residual LFX template, 50 mg of Fe_3_O_4_@MIP NPs were washed three times with 10% acetic acid. The pH of Tris buffer of the solution was adjusted at 7.0 using HCl. After washing and eluting LFX from the Fe_3_O_4_@ MIP NPs using a neodymium magnet. Then measure absorbance at zero time of 10 µg/mL LFX solution, after that Fe_3_O_4_@MIP NPs were added to the 3.0 mL of solution. The solution was then analysed by UV-Visible Spectroscopy, with the corresponding buffer used as the blank at different time interval.

Moreover, the impact of adsorbent concentration was investigated by combining 3.0 mL of LFX solution (10 µg/ mL) with 25–200 mg of Fe_3_O_4_@MIP NPs, 25 min of contact time, and pH adjusted to 7.0 For each experiment, then 0.1 mL of the a stock LFX concentration (5 mg/ mL) was transferred into 50.0 mL of tris buffer pH 7. Various amounts of Fe_3_O_4_ @MIP NPs were prepared by allowing the product to evaporate and convert into powder. These different amounts (25, 50, and 100 mg) were then examined using UV-Visible Spectroscopy to determine the optimal amount for extracting LFX from the polymer. This procedure was repeated for each amount of Fe_3_O_4_@MIP NPs under the most favourable condition.

The subsequent equation was used to determine the amount of template bound to Fe_3_O_4_@MIPs or Fe_3_O_4_@NIPs.

$${{\rm{Q}}_{\rm{T}}} = \left( {{{\rm{C}}_0} - {\rm{ }}{{\rm{C}}_{\rm{t}}}} \right){\rm{xV}}/{\rm{W}}$$

where V (L) is the initial volume of the solution, W (g) is the weight of the Fe_3_O_4_@MIPs or Fe_3_O_4_@NIPs, and C_0_ and C_t_ (mg L^-1^), respectively, are the initial and residual concentrations of LFX at time (t). Additionally, the quantity of LFX removed was estimated using the formula R% = 100 (C_0_ - C_t_) / C_0_ and reported as a percentage (R %).

### Selectivity test

Several structurally related co-administered medications, including ciprofloxacin (CPFX) and Vancomycin (VAN), were examined to assess the selectivity of LFX magnetic imprinted polymer. To conduct the study, the initial concentration of the drugs was optimized at the same concentration range for LFX, at pH 7.0 using tris buffer with the addition of 25.0 mg Fe_3_O_4_ @MIP NPs, Fe_3_O_4_ NIP NPs, and the control sample separately. An external magnetic field was used to separate the magnetic imprinted polymers and the supernatant of each medication, which was then subjected to UV-Vis spectroscopic analysis.

### Molecularly imprinted solid phase extraction of LFX from human plasma samples

A standard stock solution of LFX (1.0 mg mL^− 1^) was prepared in ACN. Spiking solutions for calibration curve and quality controls were prepared by appropriate dilution in ACN.

Briefly, 1.0 mL of plasma samples that were spiked with known variable quantities of the LFX standard solution and vortexed for 5 min in 3.0 mL ACN. To get rid of any potential interference, 2.0 mL ACN was used to precipitate plasma protein. A controlled sample was prepared by mixing 1.0 mL of double distilled water instead of plasma. The two samples were then centrifuged for 15.0 min at 2000 rpm. Then 50 mg of MIP NPs was placed with spiked plasma sample to extract LFX. After that, a magnetic field was applied externally to the solution to decant it. Furthermore, LFX was extracted from MIP NPs as described above.

## Results and discussion

### Characterization of Fe_3_O_4_@MIP NPs

**Fig. 1:**
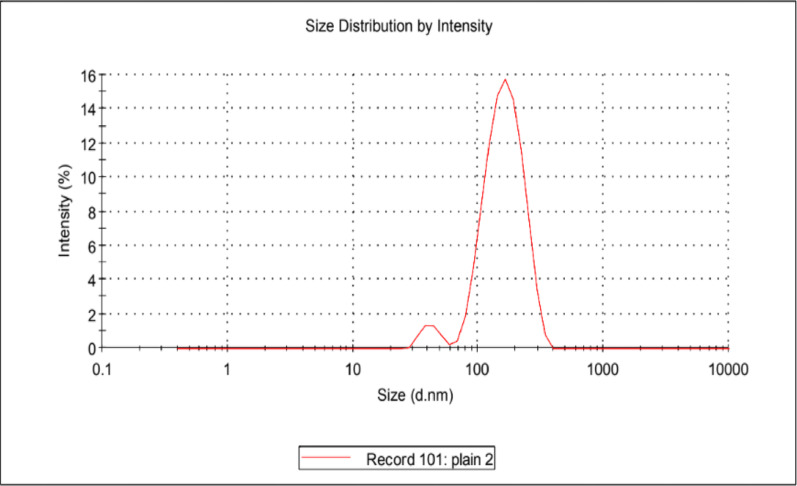
The particle size of the unmodified bare Fe_3_O_4_ NPs

**Fig. 2:**
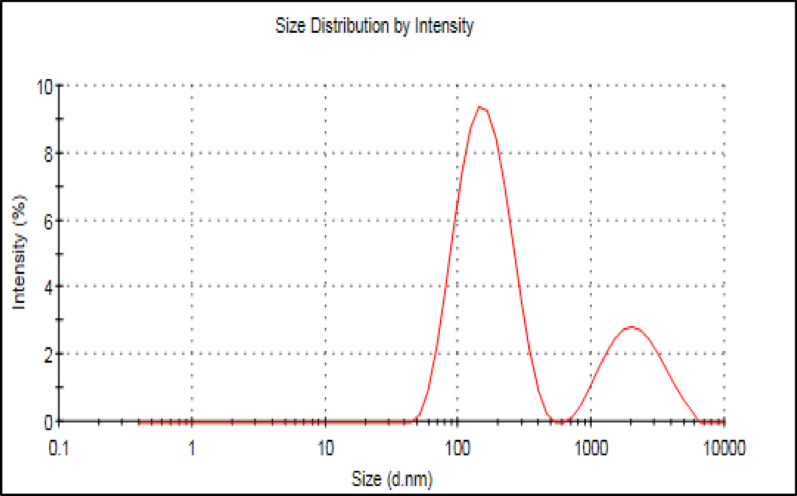
The particle size of the modified Fe_3_O_4_ @MIP NPs with LFX

The particle size of the unmodified Fe_3_O_4_ in (Fig. 1) and the modified MIP/ Fe_3_O_4_ with LFX in (Fig. 2) were examined by dynamic light scattering zeta-sizer. It’s obvious that the mean diameter of the bare Fe_3_O_4_ was about 176.9 nm as shown in (Fig. 1). However, after modifying with LFX to the diameter has approximately increased to 905 nm (over 700 nm increment) more than the actual size of the bare Fe_3_O_4_ which indicates that the polymerization process was valid and MNPs successfully coated with a thin layer that is beneficial for the imprinted template.

According to the reported poly dispersed index value in both (Fig. 1) and (Fig. 2) the heterogeneity of the sample is classified as mono dispersed particle as the number ranges from 0.1 to 0.4 which demonstrates that the difference between the large and small particles is not excessively enormous as presented in Table 1.

**Table 1:** The poly-dispersity index which measures the heterogeneity nanoparticles based on its size

		Size (d *n*.m)	% Intensity	Width (d *n*.m)
Z-Average (d.nm) 179.2	Peak 1	166.2	75.5	73.86
Pdl: 0.399	Peak 2	2247	24.5	1047
Intercept:0.922	Peak 3	0.000	0.0	0.000
Result quality: Good				

#### XPS

**Fig. 3:**
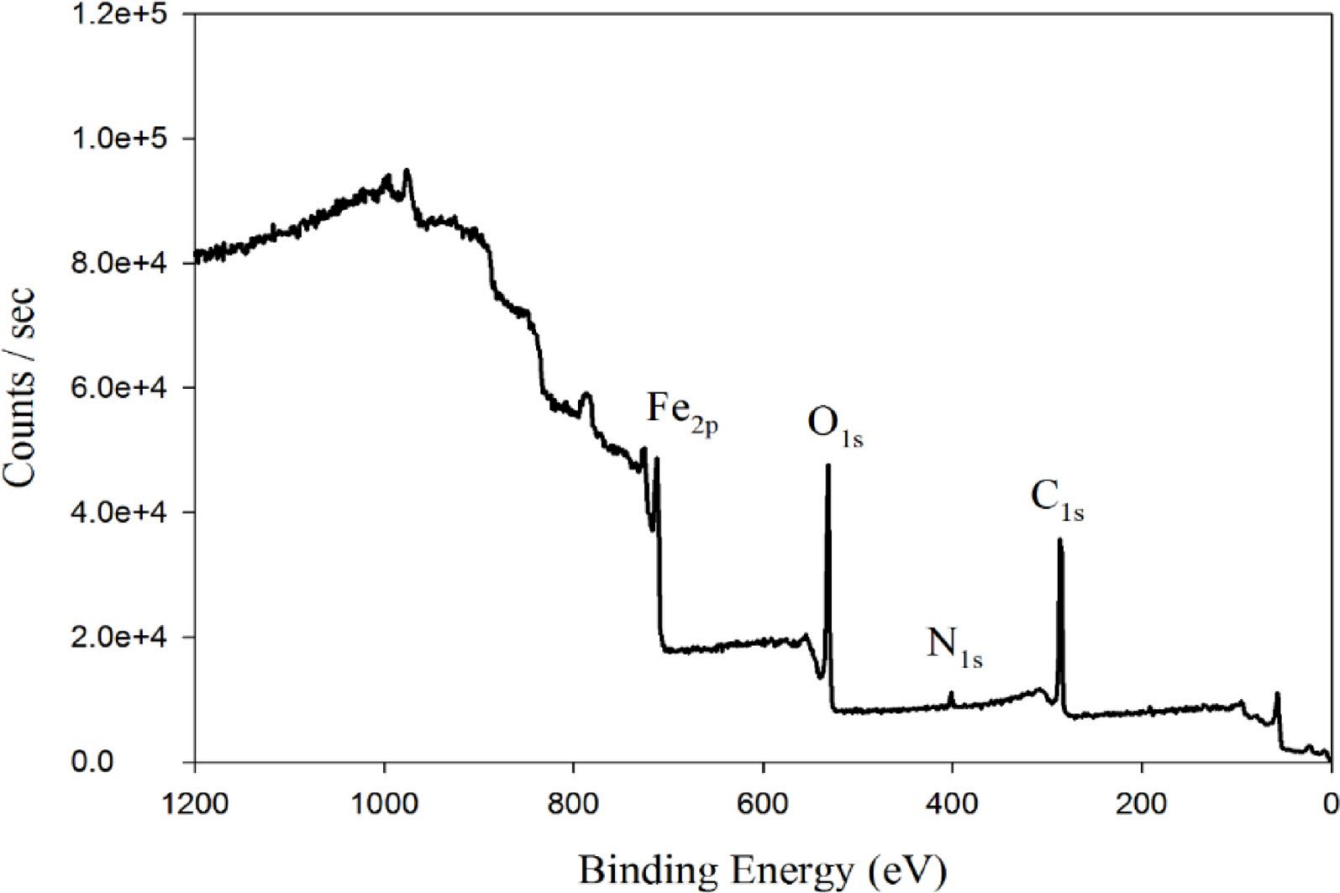
XPS of Fe_3_O_4_ @ MIP NPs

Methyldopa was auto polymerized in situ onto the surface of Fe_3_O_4_ magnetic nanoparticles, resulting in a uniform PMD coating. This polymerization process was facilitated by the alkaline medium provided by tris buffer, which promotes the formation of reactive quinone intermediates from methyldopa’s catechol groups. The successful formation of the PMD coating was confirmed by XPS (Fig. 3), which revealed characteristic peaks corresponding to Fe_2p_, O_1s_, N_1s_ and C_1s_ regions. The survey spectrum clearly showed the presence of Fe, O, N, and C elements. The Fe_2p_ peak at 711.9 eV. Similarly, O_1s_ region shows a peak around 531.2 eV. Both N_1s_ peak around 401.4 eV and C_1s_ peak around 285.8, confirm successful polymerization occurred on the surface of Fe_3_O_4_@MIP NPs.

#### SEM

**Fig. 4:**
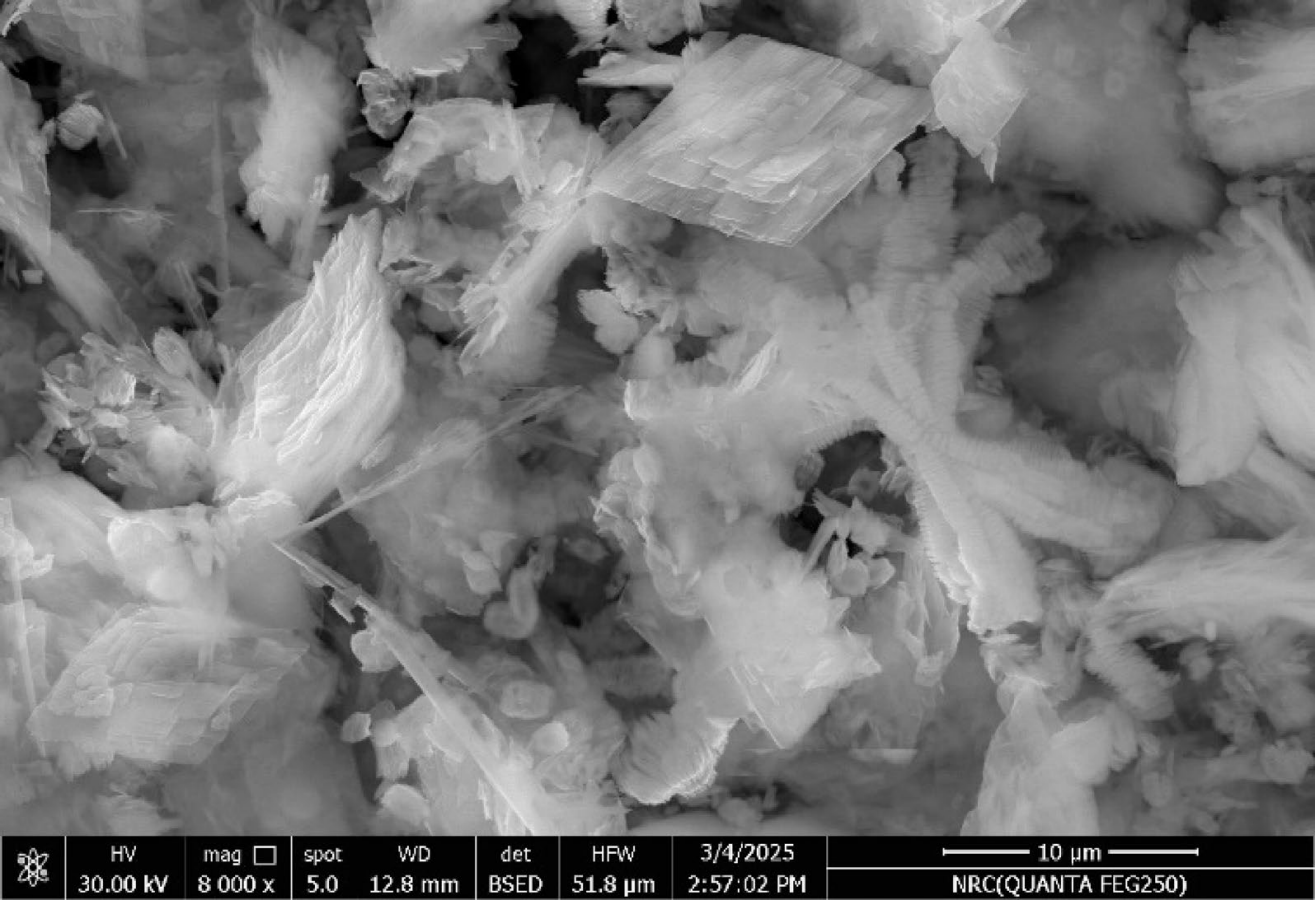
SEM image of Fe_3_O_4_@MIP NPs

The surface morphology of the Fe_3_O_4_@MIP NPs was evaluated by SEM (Fig. 4). It was observed that the surface of the Fe_3_O_4_@MIP NPs is uneven with many holes thus, confirm the successful adsorption of template molecule; LFX. The SEM image shows that the magnetic nanoparticles tend to aggregate, forming large clusters. Such aggregation is commonly observed in iron oxide nanoparticles due to strong magnetic dipole–dipole interactions. While aggregation may reduce the effective surface area and restrict adsorbate diffusion into inner sites, a significant number of functional groups remain accessible on the external surfaces. This explains the maintained adsorption performance observed in our study. Moreover, the magnetic nature of these clusters facilitates their rapid and efficient recovery from solution.

#### EDX elemental analysis

**Fig. 5:**
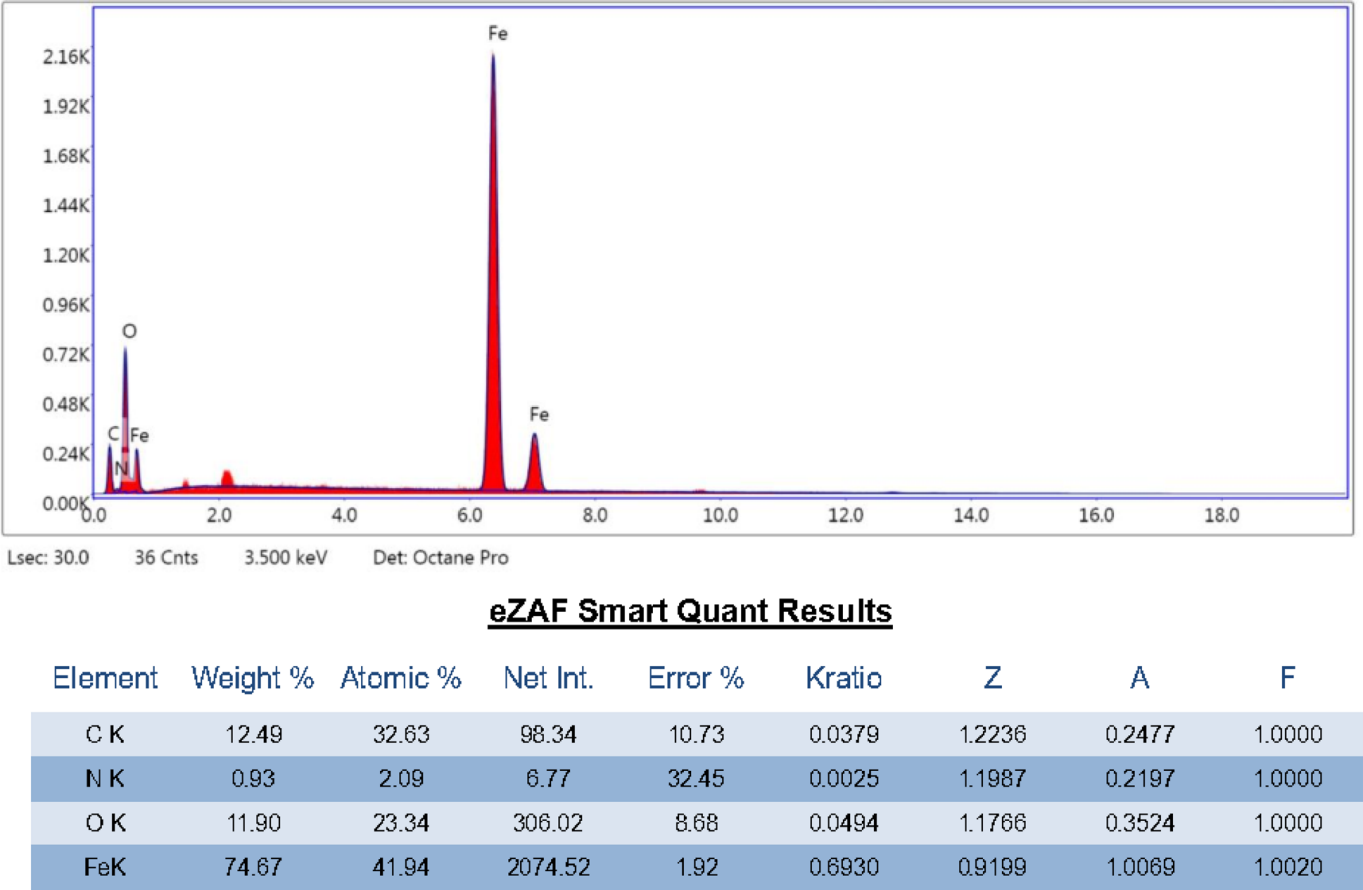
EDX of Fe_3_O_4_@MIP NPs

EDX analysis was performed to confirm polymerization took place. EDX spectrum (Fig. 5) indicated the presence of Fe, C, O, N confirming that polymerization occurred on the surface of Fe_3_O_4_@MIP NPs. The dominant Fe content (nearly 75 wt%) with significant O confirms the synthesis of iron oxide–based magnetic nanoparticles, which are responsible for their superparamagnetic or ferrimagnetic properties. The presence of C and N suggests successful surface functionalization/modification which improves dispersion, prevents aggregation, and provides active sites for adsorption or bioconjugation. The relatively high C content (12.5 wt%) supports the idea of an organic shell or stabilizer coating, which also aligns with the observed SEM aggregation (Fig. 4).

#### FTIR

**Fig. 6:**
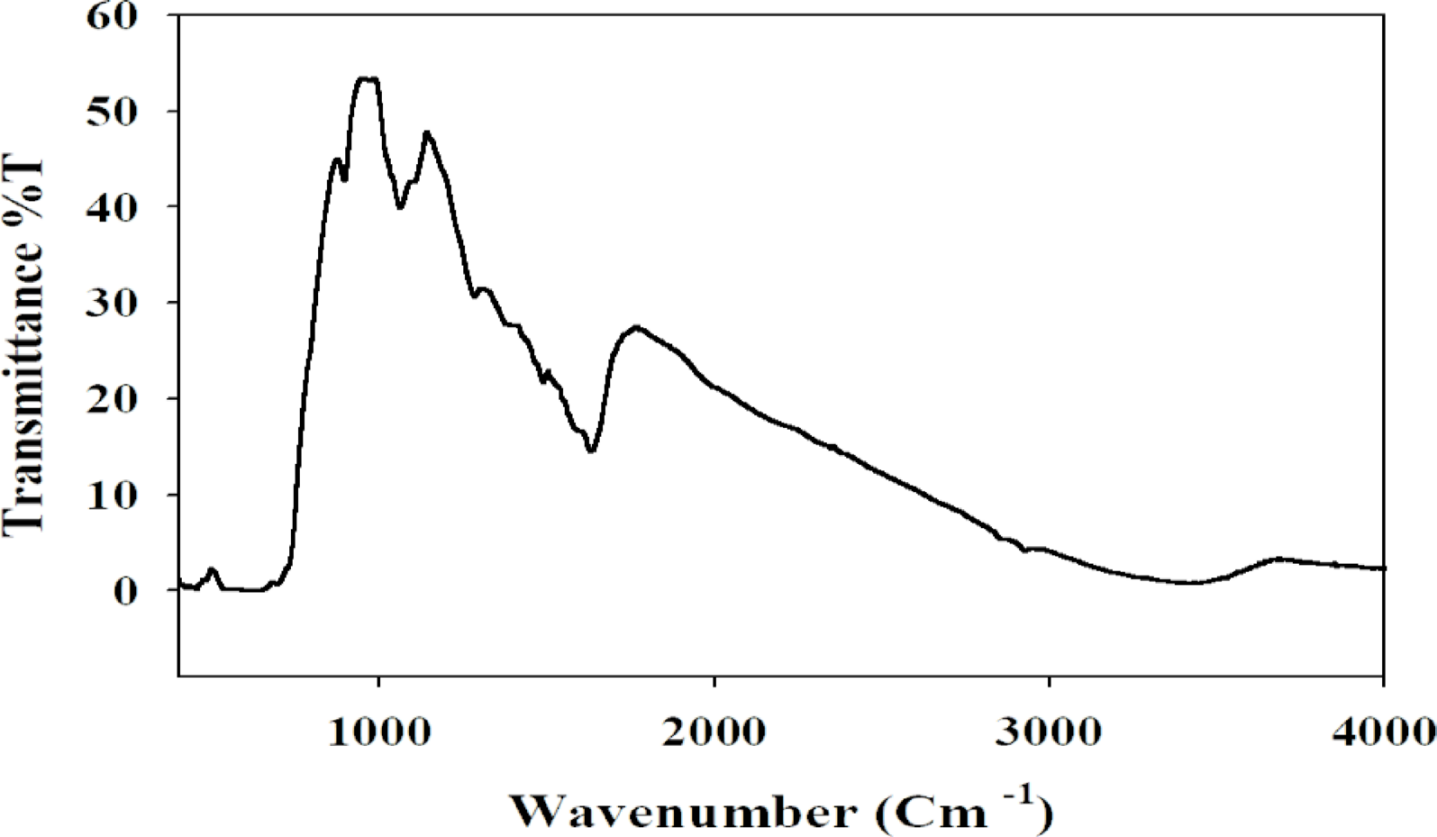
FTIR spectra of Fe_3_O_4_@MIP NPs

Careful inspection of the FTIR spectrum (Fig. 6) shows that the characteristic forked peak of the primary amino group at 3300–3500 cm⁻¹ (a key fingerprint for primary amines) typically present in the FTIR spectrum of methyldopa is absent in our FTIR. Also, characteristic sharp catechol O–H stretching vibrations and free monomer-related bands were significantly diminished or shifted in the polymer spectrum, suggesting that the methyldopa underwent successful oxidative self-polymerization during MIP formation. Furthermore, the XPS high-resolution spectrum of N_1s_ displays the expected binding energies corresponding to the imprinted polymer structure, without additional peaks that would indicate the presence of free or unreacted monomer. These results confirm that the observed bands are attributable to the polymeric MIP coating on Fe₃O₄ NPs and not dominated by unreacted methyldopa.

#### XRD

The crystallographic structure of Fe₃O₄@MIP NPs was characterized by powder X-ray diffraction (XRD), as shown in Figure S1. The diffraction pattern displayed six characteristic peaks at 2*θ* values of 30.42°, 35.78°, 43.47°, 53.89°, 57.44°, and 63.03°, which are in good agreement with the standard reference data for magnetite (JCPDS card No. 19–629). The XRD patterns also indicated that the synthesized polymeric particles are crystalline in shape.

### Analysis technique

**Fig. 7:**
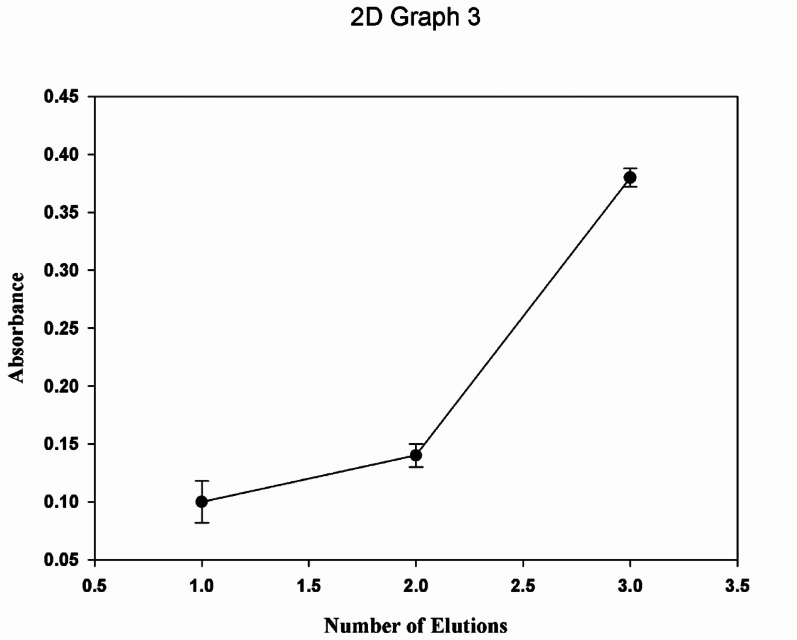
The adsorption of LFX to Fe_3_O_4_@MIP NPs after three elution with 10% acetic acid.

One of the most crucial steps that should be applied to the MIP/NPs and is particularly considered to be the rate-limiting step while measuring the MIP/Fe_3_O_4_ in UV-Spectroscopy is the washing/elution step, to ensure the complete removal of the template. To test the adsorption of LFX to the Fe_3_O_4_@MIP NPs, the drug was washed three times with 10% acetic acid to retreat the cavity of the MNP from LFX and test the adsorption of three elution that were obtained from the Fe_3_O_4_@MIP NPs. It was found that the adsorption has relatively decreased to the utterly appropriate washing technique starting from elution two which indicates that the adsorption of LFX to the polymer is justifiable, Fig. 7.

**Fig. 8:**
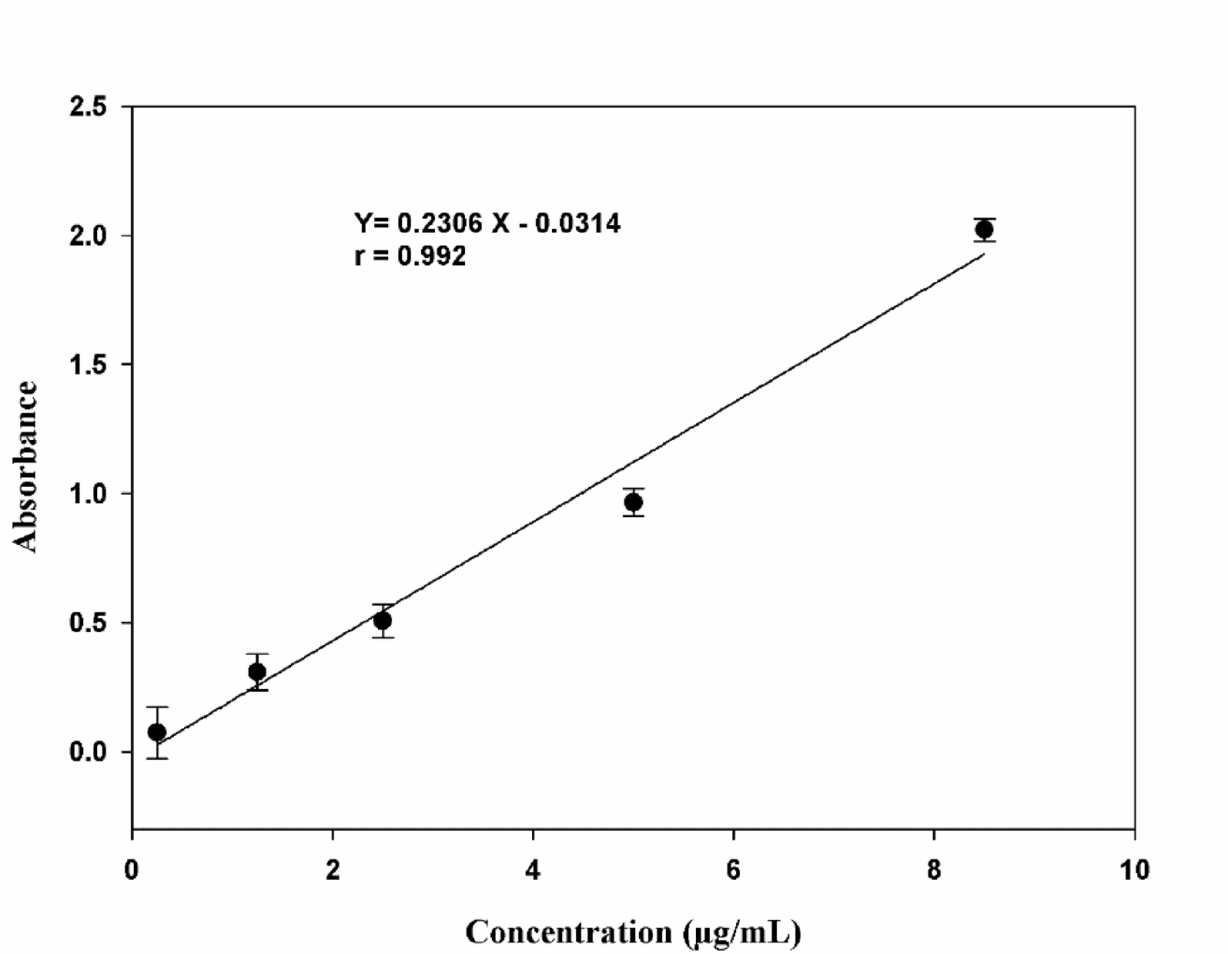
Calibration curve of LFX in Tris buffer pH 7.0

Moreover, the existential adsorption effect of LFX on the polymer was further investigated to assure the maximum adsorption of LFX. Calibration curve was constructed utilizing five different testing concentrations the peaks were found to be linear with the concentration of LFX as presented in Fig. 8.

**Fig. 9:**
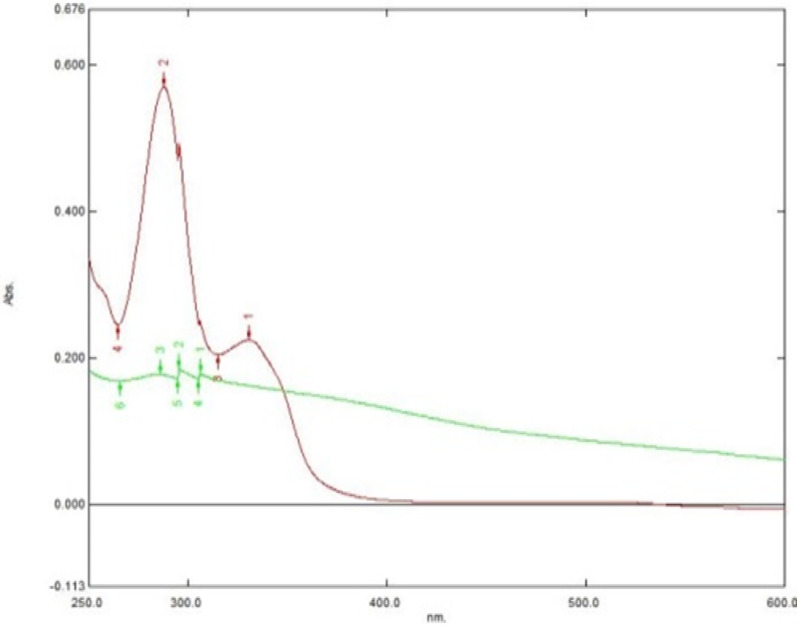
Absorption spectra of LFX (5.0 µg/mL) in phosphate buffer pH 7.0 without incubation (red line) and with incubation (green line) of Fe_3_O_4_@MIP NPs

Furthermore, the ability of Fe_3_O_4_@MIP NPs to extract LFX was additional tested by measuring the same concentration twice; one without incubation of Fe_3_O_4_ @MIP NPs and one with incubation Fe_3_O_4_@MIP NPs. It was observed that LFX with the Fe_3_O_4_@MIP NPs and phosphate buffer has almost no absorption compared to the control LFX sample indicating that the Fe_3_O_4_ @MIP has higher adsorption to the template drug, Fig. 9.

### Optimization of the molecular imprinting magnetic solid phase extraction procedure

The extraction efficiency of the prepared Fe₃O₄@MIP NPs is strongly influenced by their surface charge, stability, and adsorption characteristics. The z-potential value was measured to be − 23.5 mV using Zetasizer analysis, indicating moderate colloidal stability that is sufficient to prevent aggregation and maintain particle dispersion during the extraction process. This negative surface charge not only contributes to maintaining the accessibility of the imprinted binding sites but also enhances electrostatic interactions with levofloxacin, which exists in a zwitterionic/partially anionic form under physiological pH. Furthermore, the molecularly imprinted polymer shell provides recognition sites that are complementary in size, shape, and functionality to LFX, enabling selective adsorption through a combination of electrostatic forces, hydrogen bonding, and hydrophobic interactions. These combined effects explain the high recovery and selectivity achieved when applying the Fe₃O₄@MIP NPs sorbent for solid-phase extraction of LFX. To attain optimal conditions for extraction of the target drug; LFX, several factors including extraction time, pH, amount of adsorbent and effect of sample volume were examined and optimized to enhance LFX pre-concentration and clean-up.

#### Extraction time

**Fig. 10:**
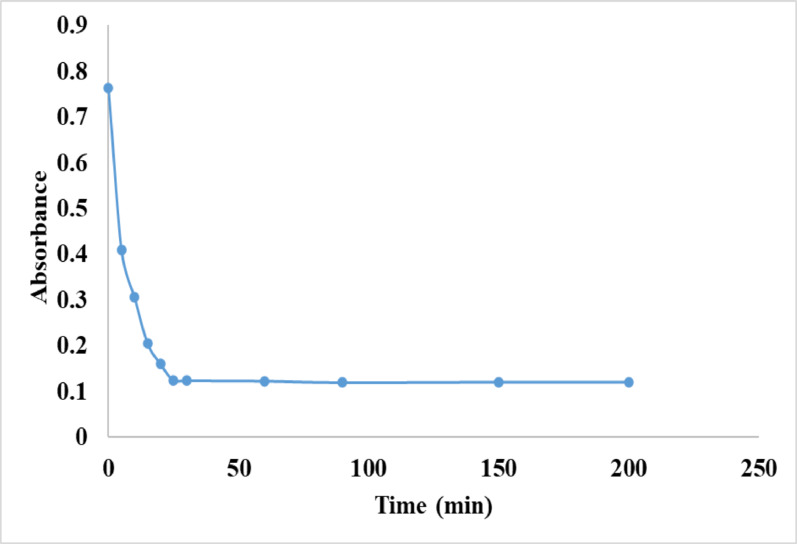
Effect of extraction time on the adsorption of LFX (6.0 µg/mL) onto Fe_3_O_4_@MIP NPs; 50 mg, using phosphate buffer (pH 7.0)

The impact of extraction time on the efficiency of LFX (6.0 µg/mL) extraction using 50.0 mg of Fe₃O₄@MIP NPs in 20 mL of ACN was evaluated over a range of 5.0 to 200.0 min. Optimal extraction was achieved within 25.0 min, as extending the contact time beyond this point did not lead to any significant improvement in extraction efficiency, Fig. 10. This plateau effect is likely due to the system reaching equilibrium on the surface of the Fe₃O₄@MIP NPs, preventing further LFX uptake from the solvent. Additionally, the relatively short extraction time of 25.0 min is considered efficient and is likely due to the excellent dispersion of the Fe₃O₄@MIP NPs in the solvent, which increases the available surface area for interaction with the target molecule, LFX. Therefore, a 25-min extraction time was selected for further experiments.

### Effect of adsorbent amount

**Fig. 11:**
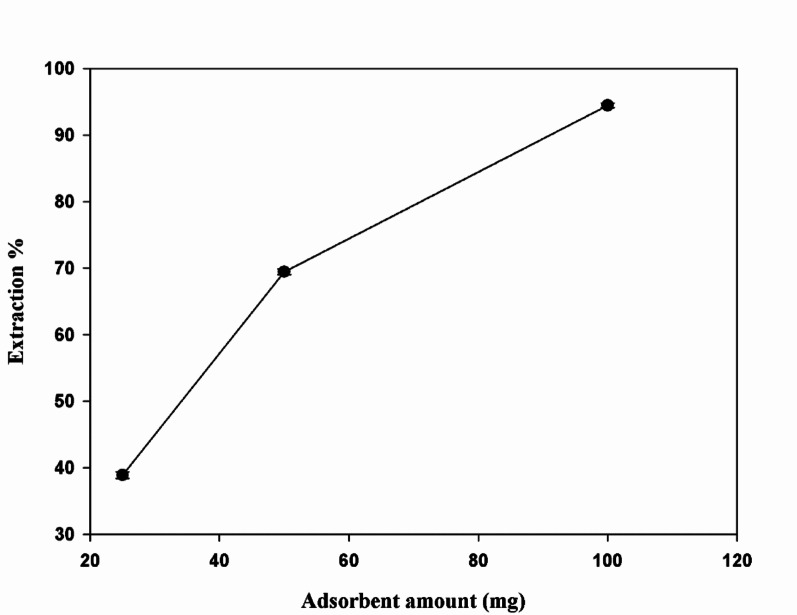
Effect of adsorbent amount of 25.0 mg, 50.0 mg, and 100.0 mg of Fe_3_O_4_ @MIP NPs on extraction of LFX

The UV-Visible Spectroscopy analysis revealed that as the amount of Fe₃O₄@MIP NPs increased (25, 50, and 100 mg), the absorbance of LFX decreased. This reduction in absorbance suggests that a higher quantity of Fe₃O₄@MIP NPs provides more accessible adsorption sites, leading to greater adsorption of LFX. The data indicate that the adsorption capacity of the Fe₃O₄@ MIP NPs enhances with increasing the amount of adsorbent. This correlation is also reflected in the extraction efficiency, which improves significantly with higher adsorbent amounts due to the more effective removal of LFX from the solution as shown in Fig. 11.

#### Effect of pH

The adsorption capacity of Fe_3_O_4_@MIP NPs for LFX was significantly influenced by the pH of the solution. Therefore, three different pH using tris buffer were tried 6,7,8. The carboxylic acid groups present in the Fe_3_O_4_@MIP NPs’ cavities exhibit a strong hydrophilic affinity, and they can form hydrogen bonds with the amine and carboxylic groups of LFX. It was revealed that the Fe_3_O_4_ @MIP NPs had the highest adsorption capacity at pH 7.0. At lower and higher pH values, the adsorption capacity was considerably reduced. Therefore, pH 7.0 was selected as the optimal condition for the adsorption of LFX onto Fe_3_O_4_ MIP NPs as it showed a 70% successful adsorption rate to the MIP NPs compared to others.

#### Effect of sample volume

To obtain an accuracy of measurement results and high sensitivity the preconcentration factor was calculated and found to be 93.5.

### Binding test

Various amounts of LFX were added in phosphate buffer to a constant weight of Fe_3_O_4_ @MIP NPs (50 mg) to assess the binding capacity of the MIP NPs. It was found that the adsorption of LFX to 50 mg MIP NPs was 86% at a concentration of 50.0 µg/mL; however, it started to decline at 200.0 µg/mL (76%) and continued until 300 µg/mL (70.3%), Table 2.

**Table 2:** The free and binding ratio of Levofloxacin against different concentrations that were tested twice one with the Fe_3_O_4_ @MIP NPs and one with the phosphate buffer alone which acts as a control sample

Concentration ( µg/mL)	Absorbance of control sample	Absorbance of MNP	Free%	Binding ratio%
50	1.358	0.161	13%	86.8%
100	1.3582	0.138	11.6%	88%
150	0.942	0.101	12%	87%
200	0.551	0.114	23%	76%
250	0.734	0.191	28%	71.8%
300	1.049	0.296	29%	70%

### Selectivity test

The extraction of LFX and its structurally similar or co-administered drugs ciprofloxacin and vancomycin was assessed to gauge the selectivity of the synthesised Fe_3_O_4_ MIP NPs. Vancomycin, a glycopeptide antibiotic, was tested for the binding of MIP NPs, and the results were analyzed after separating the supernatant from the MIP NPs by a neodymium magnet using UV-spectroscopy. Vancomycin is used to treat a variety of bacterial infections, including complicated skin infections, bloodstream infections, endocarditis, bone and joint infections, and meningitis. The initial concentration of drugs (8 µg/mL) was extracted by 25 mg of Fe_3_O_4_@MIP NPs and Fe_3_O_4_ NIP NPs at pH 7.0 using tris buffer. Results showed that the adsorption of LFX was better than all of them; however, Vancomycin showed slightly adsorption to the magnetic nanoparticles but not greater than LFX. The significance of the process and the adsorption of LFX along with its co-administered drugs was assessed by calculating the partition coefficient K using the subsequent equation:

$$ {\rm{K }} = {\rm{ Cp}}/{\rm{Cs}}$$

where Cs is the concentration of LFX still present in the solution and Cp is the quantity of LFX bound by Fe_3_O_4_MIP or Fe_3_O_4_ NIP NPs.

Additionally, the selectivity properties of Fe_3_O_4_ MIP and Fe_3_O_4_ NIP NPs toward LFX along with its subsequently delivered drugs were assessed using the imprinting factor (IF) and selectivity coefficient (SC) (ciprofloxacin and vancomycin). The following formulas were used to calculate the IF and SC.

$$ {\bf{Imprinting}}{\rm{ }}{\bf{factor}}{\rm{ }}\left( {{\bf{IF}}} \right) = {\rm{ Ki}}/{\rm{Kc}}$$

$${\bf{Selectivity}}{\rm{ }}{\bf{coefficient}}{\rm{ }}\left( {{\bf{SC}}} \right) = {\rm{ I}}{{\rm{F}}_{{\rm{Lfx}}}}/{\rm{I}}{{\rm{F}}_{\rm{i}}}$$

where Ki and Kc represent the medication’s partition coefficients for the Fe_3_O_4_ MIP and Fe_3_O_4_ NIP NPs. Moreover, IF_Lfx_ and IFi are the imprinting factors for LFX and concurrent medications, respectively. The results are presented in Table 3. It is shown that the imprinting factor for LFX indicates the available cavity sites and affinity for MIP which demonstrates the higher adsorption capacity of LFX compared to other drugs. The higher LFX number also reveals that MIP NPs is complement in size, shape, and structure to LFX which illustrates higher adsorption number of cavity sites and selectivity over the competing medicines.

**Table 3:** The partition coefficients, adsorption capacity, selectivity coefficients and imprinting factors of LFX and its co-administered drugs (CPFX and VAN) for the imprinted Fe_3_O_4_@MIP NPs and control Fe_3_O_4_@NIP NPs

Analyte	K _MIP_	K _NIP_	IF	SC
Levofloxacin	2.37	0.17	13.66	-
Ciprofloxacin	0.34	0.18	1.91	10.06
Vancomycin	0.07	0.05	1.36	7.14

### Sample processing

#### Sample preparation

Matrix components such as proteins can profoundly disrupt molecular shape selective interactions, thereby reducing extraction recoveries [40]. To minimize this effect, proteins in biological fluids were removed by simple precipitation procedures, a common approach that employs solvents such as methanol, ACN, or certain acids [40]. The aim of this study was to develop a selective procedure for the extraction of LFX from human plasma. The optimum extraction procedure was applied to plasma samples. ACN was employed as the loading solvent to achieve effective protein precipitation, while 10% acetic acid was used as the eluting solvent to obtain maximum recovery of LFX.

#### Determination of LFX in human plasma sample

A practitioner can track a patient’s dosage and get the optimum therapeutic result with the least amount of toxicities by assessing a drug’s plasma concentration. The Fe_3_O_4_@MIP NPs were successfully employed in our proposed Magnetic SPE technique to extract LFX from plasma for therapeutic drug monitoring applications. Results depicted that 93.5% of LFX was successfully extracted from the plasma sample which ensures that specificity, selectivity, and extraction of LFX by the MIP NPs. According to the FDA, the successful range of extraction for therapeutic drug monitoring is 20% [41], making LFX legally efficient for the MIP NPs; additionally, the acceptance criteria for each back-calculated standard concentration was a 7% deviation from the nominal value, except where a 20% deviation was permitted by the FDA.

#### Comparison with reported methods

To further evaluate the performance of the developed Fe₃O₄@MIP NPs, a quantitative comparison with previously reported extraction methods for fluoroquinolones was conducted. Table 4 summarizes the recovery percentages and detection methods employed in representative studies, including those focused on levofloxacin. As shown, the recovery achieved with our method is comparable to recoveries reported for other extraction techniques. This supports the robustness and efficiency of the proposed approach, particularly when considering its simplified synthesis and cost-effectiveness, which may provide advantages for routine clinical applications and therapeutic drug monitoring in resource-limited settings.

**Table 4:** Comparison of the extraction of fluoroquinolones, including Levofloxacin with other reported methods

Sorbent	Matrix	Analyte	Detection	Recovery %	Reference
Fe_3_O_4_/MCNT/MIP	Human serum	Gatifloxacin	LC-UV (325 nm)	79.1–85.3	[42]
Fe_3_O_4_/MIP	Human urine	Ofloxacin, pefloxacin, enrofloxacin, norfloxacinand gatifloxacin	LC-UV (278 and 294 nm)	83.1-103.1	[43]
Fe_3_O_4_/MCNT/MIP	Serum	Levofloxacin	LC-UV (280 nm)	78.7–83.4	[44]
Fe₃O₄@MIP NPs	Spiked Human plasma	Levofloxacin	UV–spectroscopy of (298 nm)	93.5	Our work

## Conclusion

An efficient and affordable procedure for synthesis of magnetic molecularly imprinted NPs was presented in this study. The suggested Fe_3_O_4_@MIP NPs were evaluated for extraction and determination of LFX in human plasma sample by UV-spectroscopy. Results showed an efficient and selective separation of LFX in human plasma samples with a recovery percentage of about 93.5% indicating, the special ability of Fe_3_O_4_@MIP NPs for extraction.

The presented Fe_3_O_4_ NPs were produced by surface imprinting and self-polymerization. The chosen monomer was effective and functional, and its combination with mussel adhesive properties improved adsorption and eliminated the large consumption of time using MSPE. Moreover, the suggested extraction method offered a quick, precise, and recyclable extraction with an enhanced sample clean-up capability. Eventually, the suggested Fe_3_O_4_@MIP NPs can be a crucial subistitute for typical extraction techniques for biological sample pre-concentration.

## Supplementary Information

Below is the link to the electronic supplementary material.

Supplementary Material 1

## Data Availability

The datasets used and/or analysed during the current study are available from the corresponding author on reasonable request.
