# Supplementary material for: Synthesis and characterization of core-shell mussel inspired magnetic molecularly imprinted polymer nanoparticles for the solid phase extraction of levofloxacin in human plasma
Source: BMC Chem. 2025 Oct 22;19(1):280. doi: 10.1186/s13065-025-01641-9 (PMC12548232; doi:10.1186/s13065-025-01641-9)
Supplement: Supplementary file 1 — Supplementary Material 1 [file 13065_2025_1641_MOESM1_ESM.docx]

**Supplementary Material for**

**“Synthesis and characterization of core-shell mussel inspired magnetic molecularly imprinted polymer nanoparticles for the solid phase extraction of levofloxacin in human plasma”**

Aya A. Mouhamed^*1^, Kareem Orensa^2^, Maria Osama Mekhail ^3^, Noha I. Abdelaziz^2^, Amr M. Mahmoud^1,2^, Dina A. El Mously^1^

^1^ Pharmaceutical Analytical Chemistry Department, Faculty of Pharmacy, Cairo University, El-Kasr-El Aini Street, Cairo, 11562, Egypt

^2^School of Pharmacy, Newgiza University, Km. 22 Cairo-Alex Road, Giza P.O. Box 12577, Egypt

^3^ Faculty of Pharmacy, Cairo University, El-Kasr-El Aini Street, Cairo, 11562, Egypt

*Corresponding author email: [aya.ahmed@pharma.cu.edu.eg](mailto:aya.ahmed@pharma.cu.edu.eg)


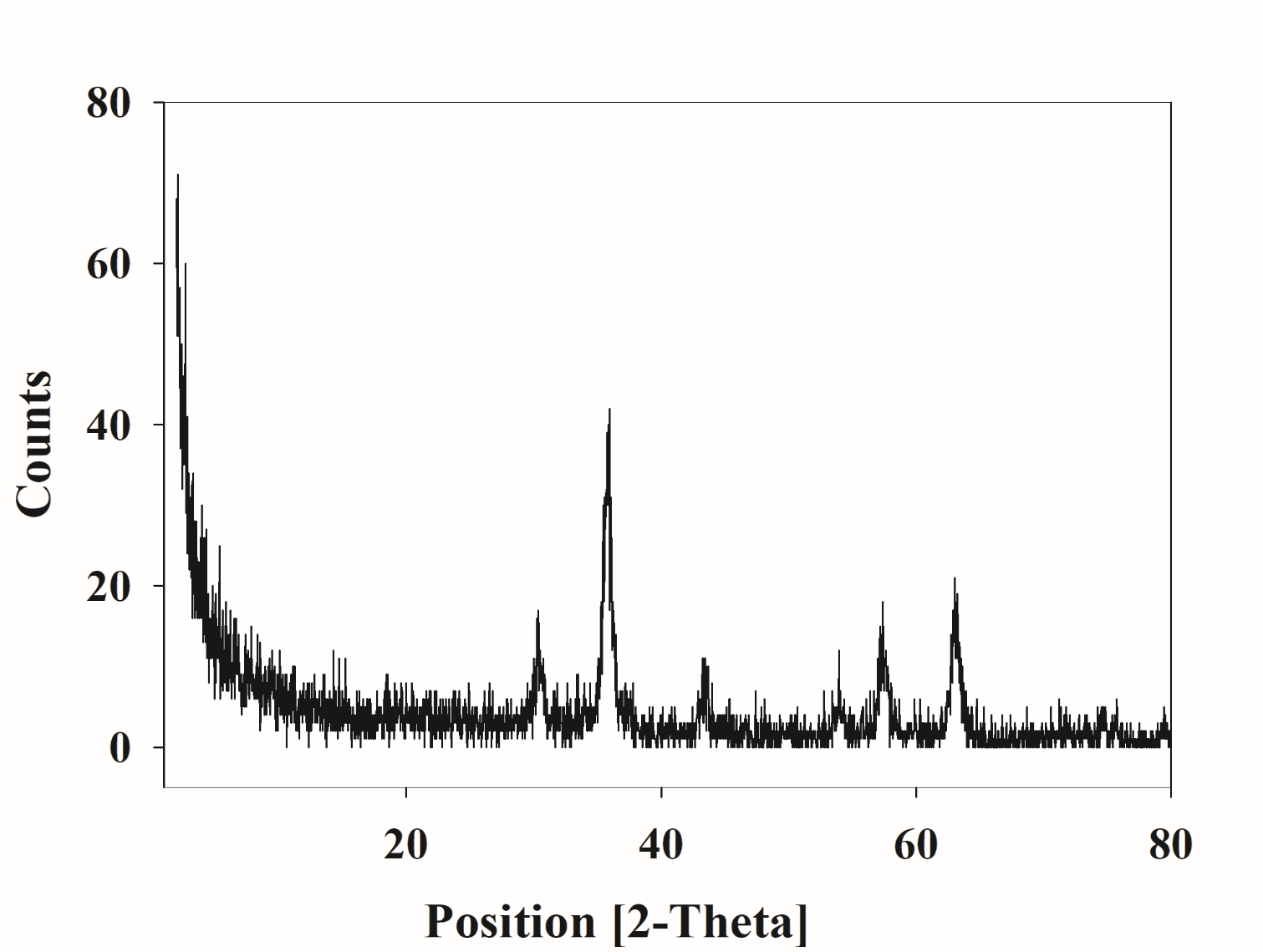


**Figure S1.** XRD pattern of Fe_3_O_4_@MIP NPs**.**
